# Immunogenicity of adalimumab in patients with noninfectious uveitis based on therapeutic drug monitoring

**DOI:** 10.1186/s12967-025-07352-y

**Published:** 2025-11-06

**Authors:** Jing-Wen Han, Yue Zhou, Jian Guo, Xuling Chen

**Affiliations:** 1 Department of Pharmacy, The First Affiliated Hospital, Fujian Medical University, Fuzhou, 350005 China; 2 Department of Pharmacy, National Regional Medical Center, Binhai Campus of the First Affiliated Hospital, Fujian Medical University, Fuzhou, 350212 China; 3 Chongqing Orthopedic Hospital of Traditional Chinese Medicine, Chongqing, 400012 China; 4 Department of Ophthalmology, The First Affiliated Hospital, Fujian Medical University, Fuzhou, 350005 China; 5 Department of Ophthalmology, National Regional Medical Center, Binhai Campus of the First Affiliated Hospital, Fujian Medical University, Fuzhou, 350212 China; 6 Fujian Institute of Ophthalmology, The First Affiliated Hospital, Fujian Medical University, Fuzhou, 350005 China

**Keywords:** Therapeutic drug monitoring, Uveitis, Adalimumab, Anti-adalimumab antibody, Immunogenicity

## Abstract

**Background:**

To evaluate the utility of therapeutic drug monitoring (TDM) in adalimumab-treated noninfectious uveitis (NIU) patients and determine the prevalence, incidence, and risk factors for anti-adalimumab antibody (AAA) development.

**Methods:**

In this retrospective cohort study, the frequency of AAA development, the association between serum drug concentrations and clinical efficacy, and the clinical utility of TDM were evaluated in NIU patients treated with adalimumab (ADA). Eligible patients were divided into a TDM group, in which serum ADA and AAA levels were measured prior to dosing and the treatment regimens were adjusted accordingly, and a control group, in which management was based on clinical judgement. Treatment responses were systematically assessed in both groups throughout the follow-up.

**Results:**

We established a therapeutic trough concentration window of 2.9–5.8 µg/mL for ADA in NIU patients, with serum trough levels exceeding 8 µg/mL suggesting a potential “ceiling effect.” Overall, patients managed with TDM achieved significantly better clinical outcomes than those without TDM: the median time to inflammation remission was shorter (28 days vs. 42 days), the recurrence rate was lower (15% vs. 42%), and the incidence of adverse events was reduced (10% vs. 31%). TDM guidance was implemented for 20 patients (51.3%), with the remaining 19 (48.7%) serving as controls without TDM guidance. Among the 20 patients managed with TDM guidance, six patients (30.0%) who demonstrated inadequate clinical response to adalimumab after at least six months of therapy were classified as nonresponders. Anti-adalimumab antibodies (AAAs) were detected in 3 patients among the nonresponders, with a median concentration of 1041 ng/mL (range: 578.1–1844.3 ng/mL). These patients presented significantly lower adalimumab trough concentrations (median: 0.55 µg/mL) than AAA-negative patients (median: 3.43 µg/mL; *p* < 0.001). After TDM guidance, the median adalimumab concentration in nonresponders increased to 1.22 µg/mL, whereas the AAA concentration decreased to 894 ng/mL (range: 0–1197.8 ng/mL). Concurrently, nonresponders demonstrated measurable improvements in key clinical outcomes, including best-corrected visual acuity, central macular thickness, and standardized ocular inflammation grading scores.

**Conclusion:**

TDM of adalimumab using practical serum sampling protocols is clinically feasible and has potential benefits for patient management. AAAs were detected in 50% of nonresponders and were associated with lower concentrations of adalimumab. This study provides the first protocolized evidence for TDM implementation for NIU, addressing a critical gap in current uveitis management guidelines.

## Introduction

Uveitis is an inflammatory condition that affects the iris, ciliary body, vitreous, retina, and/or choroid and is a significant cause of preventable blindness globally. Epidemiological studies indicate that uveitis is responsible for 10%-15% of preventable blindness cases in Western countries, with this figure increasing to 25% in developing regions [1, 2]. Aetiologically, uveitis is divided into infectious and noninfectious subtypes, with noninfectious uveitis (NIU) being the most prevalent, accounting for 41%-55% of cases worldwide [3]. The management of uveitis remains clinically challenging because of its complex aetiology and the potential for vision-threatening complications, including cataracts, glaucoma, macular oedema, retinal detachment, optic nerve damage, and irreversible vision loss [4].

Corticosteroids remain the first-line therapy for NIU; however, their long-term use is limited by significant adverse effects [5]. According to the Fundamentals of Care for Uveitis (FOCUS) Initiative recommendations on noncorticosteroid systemic immunomodulatory therapy for NIU, biologic therapy is generally considered when ocular disease is inadequately controlled by corticosteroids and conventional immunosuppressants [6]. The therapeutic landscape has advanced substantially with the introduction of adalimumab (ADA), which received European Medicines Agency approval in 2016 and UK National Institute for Health and Care Excellence endorsement in 2017 for adult patients with noninfectious intermediate, posterior, and panuveitis [7], followed by approval in China in 2020 [8]. Supported by robust clinical evidence and incorporated into multiple international guidelines [9], ADA is the only FDA-approved systemic noncorticosteroid therapy for NIU and represents a major milestone in the management of this challenging condition [10].

ADA is a humanized monoclonal antibody that is specific for tumour necrosis factor alpha (TNF-α) and has shown efficacy in treating ocular inflammation, thereby reducing the risk of visual impairment in patients with uveitis [11, 12]. Although clinical trials and real-world studies have generally demonstrated favourable outcomes for ADA in the treatment of NIU, the drug’s effectiveness may not be sustained indefinitely. Pachon-Suarez et al.‘s systematic review and meta-analysis revealed that among patients with NIU who were treated with ADA, the overall prevalence of AAAs was 9%, and this figure increased to 27% by the 12th month of treatment. In addition, the incidence of AAA is significantly greater in real-world settings than in clinical trials [13]. These observations indicate that an immune response to ADA may develop in a subset of patients who receive prolonged treatment, potentially leading to reduced effectiveness or even treatment failure.

Consequently, an increasing number of individuals have advocated for the implementation of therapeutic drug monitoring (TDM) to increase the biological efficacy, safety, and cost-effectiveness of biologics. Liau et al. conducted a review of TDM for biologics in the context of psoriasis [14]. Sejournet et al. reported that TDM can be used to guide the management of patients with chronic, NIU who are treated with ADA [15]. Yao et al. analysed the application of TDM for inflammatory bowel disease (IBD) management [16]. TDM is particularly useful in evaluating primary or secondary nonresponders, adjusting dosages for patients with low drug concentrations, and monitoring patient compliance with treatment. However, TDM has not yet been routinely implemented in most ophthalmology centres, and evidence regarding the impact of ADA monitoring on treatment outcomes of NIU remains limited. In addition, because of variations in assay methodologies, patient populations, and study designs, no consensus has been reached on the optimal therapeutic concentration range or TDM-guided strategies for NIU patients [13, 17].

To address these gaps, we conducted a retrospective cohort study in NIU patients treated with ADA, aiming to evaluate the levels and incidence of AAAs, characterize serum ADA concentrations and their association with clinical responses, and preliminarily assess the clinical utility of TDM in this setting. We identified a therapeutic trough concentration window of approximately 2.9–5.8 µg/mL, with serum levels above 8 µg/mL suggesting a potential ceiling effect, and demonstrated that TDM-guided management, compared with empirical treatment adjustment, was associated with faster inflammation remission, reduced relapse rates, and fewer adverse events.

## Methods

### Study population

This single-centre, retrospective study was conducted at the Ophthalmic Center of the First Affiliated Hospital of Fujian Medical University. All patients who treated with ADA for NIU between January 2022 and December 2024 were included. The inclusion criteria were as follows: (1) Patients with NIU of any anatomic subtype (anterior, intermediate, posterior, or panuveitis) were eligible if they initiated ADA therapy owing to corticosteroid dependence, intolerance, or inadequate response to systemic corticosteroids and/or conventional immunosuppressants [18–20]. (2) Patients who were followed up for at least six months following initial treatment with ADA met the criteria. (3) Patients whose serum levels of AAAs and ADA were monitored at least twice were included. The exclusion criteria were as follows: (1) patients with a follow-up time < 6 months; and (2) patients whose diagnosis and treatment information was incomplete. All human serum samples were analysed in strict accordance with a protocol approved by the ethics committee of the First Affiliated Hospital of Fujian Medical University, China (project MRCTA, ECFAH of FMU [2024] 525). This study adhered to the principles of the Helsinki Declaration, and informed consent was obtained from both patients and their legal guardians.

In patients with bilateral uveitis, disease activity was determined on the basis of the clinical activity observed in either eye. To eliminate potential intereye correlation bias, only one eye per patient was included in the analysis. The eye with greater inflammation severity at the time of enrolment was preferentially selected. In cases where the inflammation severity was identical in both eyes according to the SUN criteria, the right eye was chosen for standardization of the analysis.

ADA (Humira, AbbVie, Inc., North Chicago, IL, USA) was administered subcutaneously. Adult patients were treated with the standard regimen, which consisted of an initial loading dose of 80 mg followed by 40 mg administered every two weeks. Paediatric dosing was adjusted according to body weight; because all paediatric patients enrolled in this study weighed ≥ 30 kg, they received 40 mg subcutaneously every other week in accordance with the approved regimen. All patients received concomitant oral corticosteroids during ADA therapy. In addition, 20 patients (51%) were treated with concomitant immunosuppressive agents, including mycophenolate mofetil (MMF), azathioprine (AZA), cyclosporine (CSA), tacrolimus, and conventional disease-modifying antirheumatic drugs (cDMARDs), such as methotrexate. For patients in the TDM group, blood samples were collected within 4 h prior to the next administration of ADA, and serum levels of ADA and AAAs were simultaneously measured.

### Examination methods and data collection

The baseline levels of uveitis activity were similar in both patient groups. Ophthalmological assessments were performed at baseline and every two weeks after the initiation of treatment. Ocular assessments included best-corrected visual acuity (BCVA) tests using an international standard logarithmic chart, noncontact intraocular pressure measurement, slit lamp biomicroscopy, and fundus examination. Anatomical classification and grading of inflammatory activity were performed according to the Standardization of Uveitis Nomenclature (SUN) Working Group criteria [21]. Anterior chamber (AC) cells were graded using the SUN classification, and vitreous haze was categorized using the National Eye Institute system [22]. All ocular assessments were performed independently by two experienced ophthalmologists, with good interobserver agreement (kappa = 0.82 for AC cell grading; kappa = 0.79 for vitreous haze).

For macular assessment, all participants underwent imaging with a swept-source optical coherence tomography angiography (SS-OCTA) system (VG200, SVision Imaging Ltd., Luoyang, China), which features a 1050 nm wavelength scanning laser. Fluorescein angiography (FFA) was routinely performed on all patients with posterior segment involvement, as defined by the SUN Working Group criteria, to evaluate retinal vasculitis and quantify vascular leakage abnormalities. Indocyanine green angiography (ICGA) was also conducted when multimodal imaging indicated features suggestive of choroidal pathology, enabling a quantitative assessment of choroidal vascular permeability and topographic mapping of stromal inflammatory foci. In cases of bilateral uveitis, the eye with more severe inflammation was designated the study eye because of a stronger correlation with clinical management decisions.

Comprehensive clinical data, including demographic characteristics such as sex, age and ethnicity, as well as medical history covering diagnosis, disease duration, relapse frequency, laterality, prior medications, adverse drug reactions, dosing frequency and concomitant use of oral corticosteroids or immunosuppressants, were collected. Relapse was defined according to the criteria used in the PSV-FAI-001 phase III study as follows: an increase of at least two grades in anterior chamber cells, an increase of two or more grades in vitreous haze, a reduction of BCVA by 15 letters or more, or the requirement for additional anti-inflammatory treatment [21, 23]. Ophthalmic history such as previous ocular interventions was documented, and uveitis activity was monitored throughout ADA treatment. The use of immunosuppressive agents, surgical history involving both ocular and systemic procedures, local and systemic complications, and treatment adjustments during therapy, such as dose modifications and treatment interruptions, were systematically documented.

### Clinical response

Clinical responses were categorized as complete response (CR), partial response (PR), or nonresponse (NR) according to ophthalmic assessment and multimodal imaging, following the definitions of Cordero-Coma et al. [24]. A CR was defined by the presence of grade 0 cells in both the anterior and posterior segments and the absence of any additional intraocular inflammation signs on ophthalmologic, OCT, and FA evaluations. A PR was identified by any of the following changes from baseline or the preceding visit: (1) a two-step decrease in Standardization of Uveitis Nomenclature grading (e.g., anterior chamber cells, vitreous haze) or reduction to grade 0; (2) resolution of cystoid macular oedema, indicated by a central retinal thickness < 300 μm without intra- or subretinal fluid on OCT; or (3) absence of retinal leakage on FA. An NR was defined as ongoing intraocular inflammation not fulfilling the criteria for a PR or CR.

Nonresponders were further classified into primary and secondary nonresponders. Previous studies have suggested that for the treatment of NIU with ADA, the initial assessment should occur at 3 months, and the final decision should be made at 6 months [6]. In our study, primary nonresponders were defined as individuals with persistent intraocular inflammation within the first three months of ADA treatment. Secondary nonresponders were identified by the recurrence of intraocular inflammation signs following an initial partial or complete response that lasted at least 3 months [25]. Previous studies have reported ADA trough concentration thresholds for nonresponse in NIU ranging from 2.7 to 7.95 µg/mL [26–28]. These variations, largely attributable to differences in assay methodologies, patient populations, and study designs, have resulted in inconsistent definitions of the optimal range. Therefore, we also investigated a therapeutic window specific to our cohort by integrating adalimumab trough concentrations with the clinical response. All patients were systematically monitored for treatment outcomes and followed for at least 6 months.

In our study, both responders and nonresponders were permitted to use a defined dosage of either systemic corticosteroids or topical steroid eye drops as part of their treatment regimen. The use of concomitant steroids was carefully documented and accounted for in our outcome analyses.

### Measurement of serum ADA and AAA concentrations

Serum samples for quantification of ADA trough levels and AAA levels were collected under three clinical scenarios: (1) during recurrence of active intraocular inflammation, (2) throughout sustained inflammatory phases in treatment-refractory patients (persisting for ≥ 3 months), and (3) during the 6-month poststabilization phase in treatment-responsive patients, prior to scheduled maintenance injections, for standardized monitoring. Five millilitres of venous blood was obtained immediately before the scheduled subcutaneous administration of ADA for all the subjects. Following centrifugation, serum aliquots were cryopreserved at -80 °C in polypropylene vials until the time of batch analysis. Quantitative determinations of free ADA concentrations were conducted using validated ELISA kits (LabCorp, Calabasas, CA, USA) according to the manufacturer’s protocols. Quantitative determinations of AAA concentrations were conducted using validated ELISA kits (Biogradetech, California, USA) according to the manufacturer’s protocols. All samples were analysed in duplicate to ensure that the measurements were reproducible.

### Implementation of TDM and interventions

**Fig. 1:**
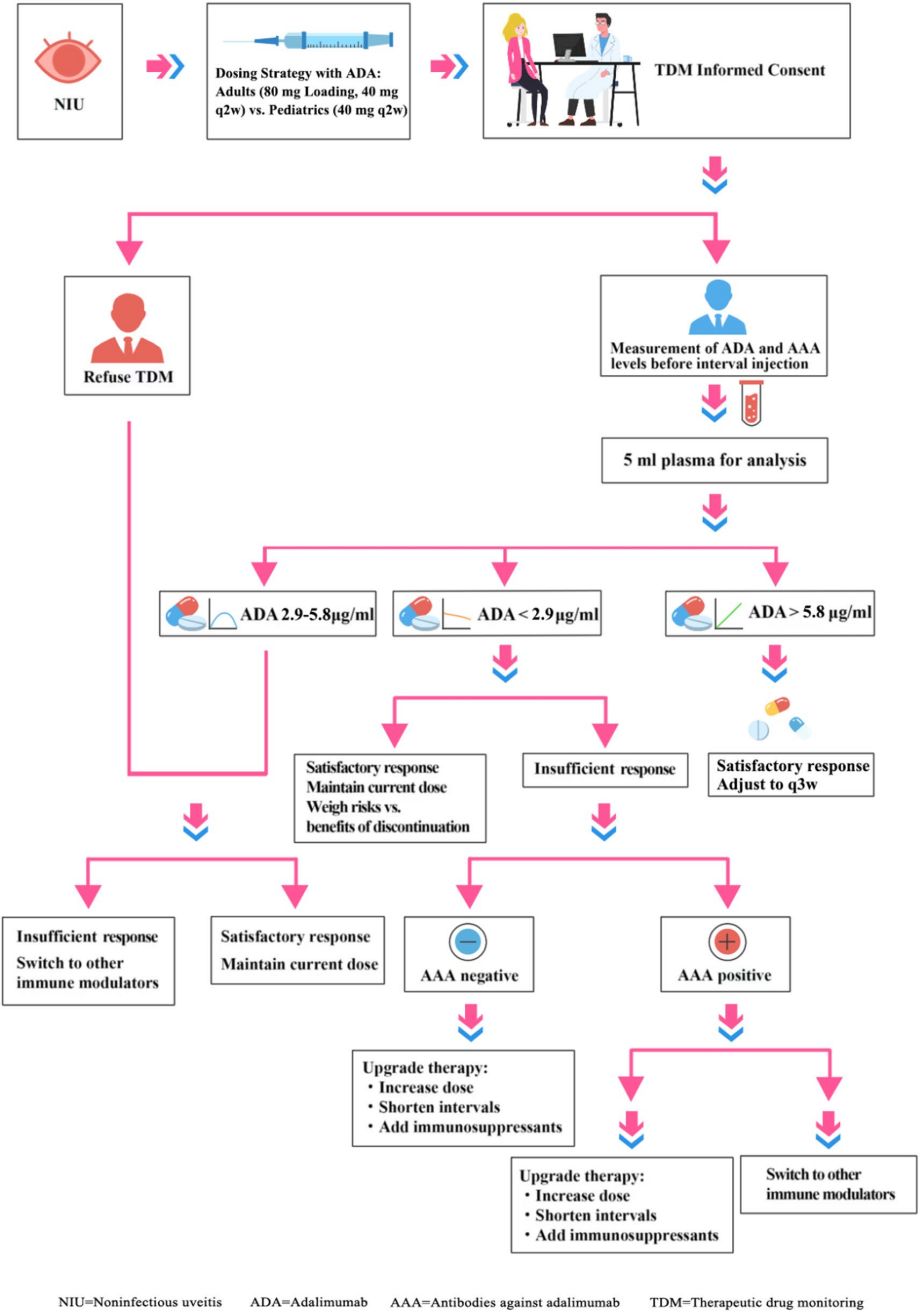
Flowchart of the study population

Once the serum concentration of ADA was measured, the patient received TDM-guided management (Fig. 1). We examined the implementation status of TDM for all eligible patients and compared the clinical and demographic characteristics of patients who received TDM with those of patients who did not.

On the basis of previous studies, the proposed therapeutic window for serum adalimumab trough concentrations in NIU ranges from approximately 2.70 to 7.95 µg/mL [26–28]. Because of variations in assay methodologies, patient populations, and other confounding factors, the optimal thresholds reported in the literature are inconsistent. Therefore, we sought to define an optimal therapeutic range tailored to our study cohort by integrating clinical responses with adalimumab trough levels. Using receiver operating characteristic (ROC) curve analysis, the therapeutic range for adalimumab trough concentrations in our population was determined to be 2.9–5.8 µg/mL. The detailed methodology and results are provided in the sections below. On the basis of this range and corresponding clinical responses, a practical clinical guidance protocol for adalimumab administration was developed. For patients with an unsatisfactory response and trough ADA levels less than 2.9 µg/mL, we recommend increasing the drug dose, shortening the dosing interval, or adjusting immunosuppressant regimens, such as cDMARDs. Some patients who develop AAAs against a primary TNFi can regain inflammatory control after switching to an alternative TNFi [29–32]. Therefore, if a patient tests positive for AAAs, we recommend switching to a non-TNF-α inhibitor or another type of TNF-α inhibitor. If a patient’s serum ADA concentration is between 2.9 µg/mL and 5.8 µg/mL and the clinical response is unsatisfactory, the recommended adjustment is to modify the immunosuppressant therapy regimen. If a patient’s serum ADA concentration exceeds 5.8 µg/mL and the clinical response is satisfactory, the recommended adjustment is to administer the treatment once every three weeks (q3w).

### Statistical analysis

Stata 13.1 statistical software (StataCorp, College Station, TX) was used for statistical analysis. Continuous variables are represented as the mean ± standard deviation (SD). The Pearson chi square test or Fisher’s exact probability method was used. Quantitative data are expressed as the mean ± standard deviation. If the data at the same time point during the treatment process between two groups followed a normal distribution, an independent samples t-test was used. Levene’s homogeneity of variance test was used to determine the homogeneity of variance for T tests results comparing two related samples; if the variance was not equal, an approximate t-test was used. Non-parametric testing was used for data that did not follow a normal distribution. The Mann–Whitney Test for two independent samples and the Wilcoxon Signed Rank test for two correlated samples were used, and statistical significance was defined as a value of *P* ≤ 05.

The trough concentrations of adalimumab in patients with complete response (CR), partial response (PR), and nonresponse (NR) were presented as median values with corresponding minimum and maximum ranges. Comparisons of trough concentrations among the three clinical response groups were performed using the nonparametric Kruskal–Wallis test. When the overall test was significant, pairwise post hoc comparisons between groups were performed. To account for multiple comparisons, the significance threshold was adjusted to ≤ 0.017 (0.05/3 pairwise comparisons) using the Bonferroni correction. Comparisons of the area under the ROC curves (AUC) were conducted using the roccomp command in Stata 13.1.The comparison of areas under the receiver operating characteristic (ROC) curves (AUCs) was performed using the *roccomp* command in Stata version 13.1, and cutoff values for treatment response were determined based on ROC curve analysis.

## Results

### Patient characteristics

The study cohort consisted of 39 patients with NIU, comprising 16 females (41%) and 23 males (59%). The anatomical subtypes were distributed as follows: 34 patients (87.2%) with panuveitis, 5 patients (12.8%) with anterior uveitis, and 1 patient each (2.6%) with intermediate and posterior uveitis. Systemic manifestations were observed in 24 patients (61.5%), whereas isolated ocular involvement was observed in 15 patients (38.5%). The distribution of systemic diseases included spondyloarthritis (*n* = 9, 23.1%), Vogt–Koyanagi–Harada disease (VKH, *n* = 5, 12.8%), Behçet’s disease (*n* = 5, 12.8%), juvenile idiopathic arthritis (JIA, *n* = 4, 10.3%), and psoriasis (*n* = 1, 2.6%). TDM was implemented for 20 patients (51.3%), with the remaining 19 (48.7%) serving as controls without TDM guidance. The average (SD) age at diagnosis was 32.8 ± 15.0 years (range, 13–60 years). The average (SD) time from the initiation of ADA treatment to laboratory testing was 33.1 ± 28.4 months (range: 6–126 months). Comprehensive demographic comparisons among the overall cohort, the TDM group, and the non-TDM group are presented in Table 1.

**Table 1:** Summary of demographic data, clinical features, and treatment information for all included patients

Parameter			All Included Patients, No. (%)	TDM-Guided Group, No. (%)	Non-TDM-Guided Group, No. (%)	*P* value
Mean age (range), yr			32.67 (8–60)	34.6 (13–60)	30.6 (8–60)	0.43
Gender	Male		23 (59)	11 (55)	12 (63)	0.748
	Female		16 (41)	9 (45)	7 (37)	-
Uveitis type by location	Anterior		5 (12)	4 (20)	1 (5)	-
	Panuveitis		34 (83)	16 (80)	18 (85)	-
Laterality	Unilateral		16 (41)	8 (40)	8 (42)	-
	Bilateral		23 (59)	12 (60)	11 (58)	-
Associated disease	No		15 (38)	9 (45)	6 (32)	-
	Yes		24 (62)	11 (55)	13 (68)	-
		Psoriasis	1 (4)	0 (0)	1 (8)	-
		SpA	9 (37)	6 (30)	3 (23)	-
		BD	5 (21)	3 (15)	2 (15)	-
		VKH	5 (21)	1 (5)	4 (31)	-
		JIA	4 (17)	1 (5)	3 (23)	-
CME before adalimumab	Yes		24 (62)	12 (60)	12 (63)	1.00
	No		15 (38)	8 (40)	7 (37)	-
Vasculitis before adalimumab	Yes		8 (21)	4 (20)	4 (21)	1.00
	No		31 (79)	16 (80)	15 (79)	-
Combination of Immunosuppressive treatment	MTX		20 (51)	9 (45)	11 (52)	-
	CsA		4 (10)	3 (15)	1 (5)	-
	MMF		13 (33)	6 (30)	7 (33)	-
	No treatment		2 (5)	2 (10)	0 (0)	-
Combined use of topical drops	Pranoprofen		39 (100)	20 (100)	19 (100)	-
	Prednisolone		39 (100)	20 (100)	19 (100)	-
	Tropicamide		33 (85)	18 (90)	15 (79)	-
	Tacrolimus		1 (3)	1 (5)	0 (0)	-
	TobraDex		28 (72)	13 (65)	15 (79)	-
Number of steroid-dependent ≧ 10 mg				8(40)	13(68)	0.111
Mean drug types (range)				3.65(2–6)	3.16(2–7)	0.275
The mean frequency of adjusting the treatment plan (range)				2.55(1–4)	1.63(0–5)	0.012
The average number of days for inflammation relief (range)				398.15(92–801)	567.1(165–980)	0.035
Mean number of relapses (N)				1.25(25)	2.37(45)	< 0.01
Mean adverse events (N)				1(20)	2(38)	< 0.01

CME = cystoid macular oedema, CsA = cyclosporine A, JIA = juvenile idiopathic arthritis, MMF = mycophenolate mofetil, MTX = methotrexate, SpA = spondyloarthritis, BD = Behcet’s disease VKH = Vogt‒Koyanagi‒Harada syndrome, TobraDex = Tobramycin and dexamethasone eye ointment. P-values were calculated using Student’s t test or Fisher’s exact test

### Levels of ADA and antibodies against ADA

**Fig. 2:**
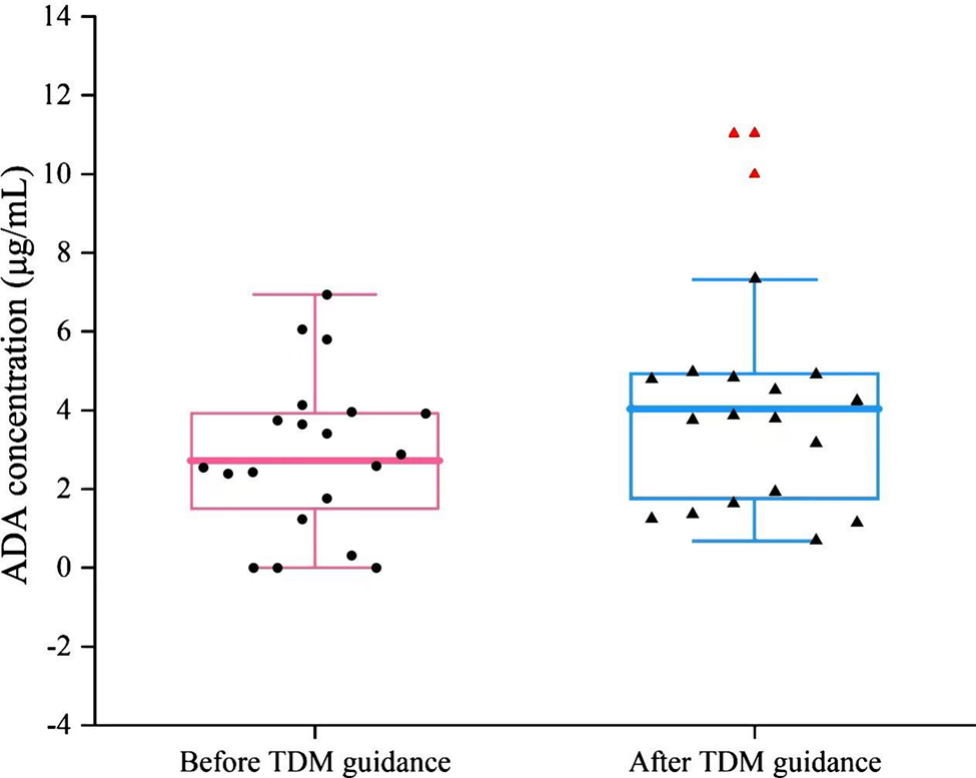
ADA concentrations of 20 patients before and after TDM guidance

Among the 39 patients receiving ADA treatment, 20 who underwent TDM (i.e., whose serum ADA concentration was measured) were managed according to TDM guidance. During the follow-up period, a total of 14 (70%) participants responded. Another six patients were identified as nonresponders, with a median time of no response of 18 weeks (range 12–24 weeks). As shown in Fig. 2, the average serum concentration of ADA before TDM guidance for 20 patients was 2.88 ± 1.95 µg/mL (ranging from 0 to 6.94 µg/mL). AAAs were detected in three out of the six nonresponders (50%), with a concentration of 1041 ± 570 ng/mL. Upon receiving TDM, the average drug concentration among the 20 patients was 4.5 ± 3.07 µg/mL (ranging from 0.0682 to 11.036 µg/mL), and the AAA concentration in the three nonresponders was 894 ± 304 ng/mL. The serum levels of ADA significantly increased (*P* < 0.0001), whereas the concentration of AAAs significantly decreased in nonresponders (*P* = 0.34).

**Fig. 3:**
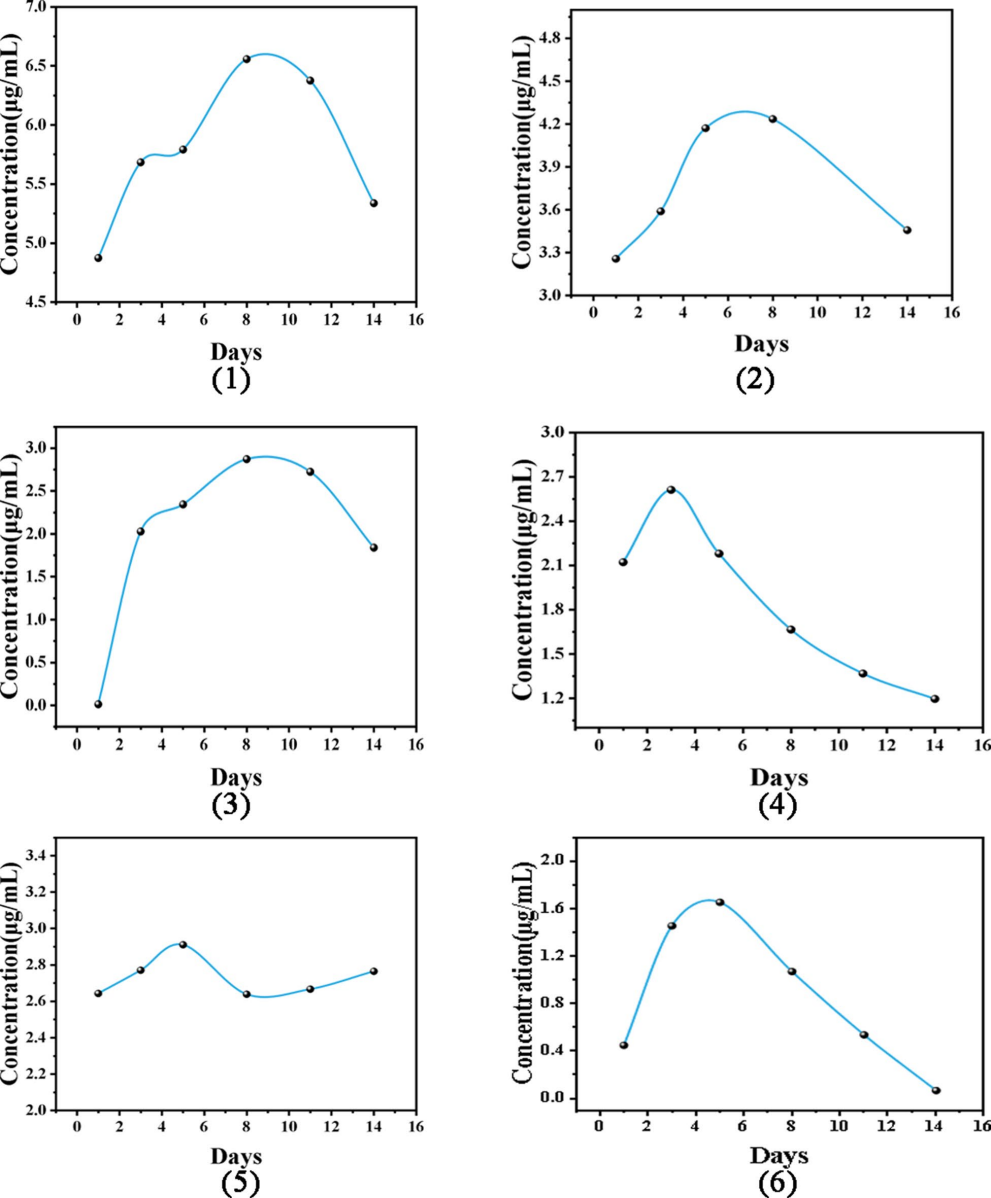
Drug concentration‒time curves of 6 nonresponders

The dynamics of ADA concentrations in 6 nonresponders within 14 days following a single dose are depicted in Fig. 3. We observed that two patients (Fig. 3a and b) with peak concentrations of ≥ 4.2 µg/ml (on days 4–6 post-dose) exhibited significant improvements in inflammation markers (for example, the anterior chamber cell grade decreased from 3 + to 0, and the central macular thickness (CMT) was reduced by ≥ 30%). Another patient (Fig. 3c) with a lower peak (2.9 µg/ml on day 5) still achieved a reduction in CMT from 450 μm to 320 μm, with an anterior chamber inflammation resolution time 50% shorter than that of other patients with low ADA concentrations. Additionally, AAAs were detected in one nonresponder after only 4 weeks of treatment with ADA. We adopted an upgraded therapy that reduced the dosage interval from 2 weeks to 1 week. However, the average trough concentration was still only 0.746 µg/mL after 7 rounds of blood drug concentration monitoring, which is far below the recommended treatment threshold. Simultaneously, the clinical response was persistently insufficient (BCVA decrease ≥ 0.2 logMAR, aqueous humour cell count > 1+), ultimately necessitating a switch to IL-6 inhibitors for treatment.

Among the three nonresponders identified as having elevated serum AAA levels, one was a paediatric patient who could not tolerate the adverse reactions associated with MMF. Therefore, we shortened the dosing interval. Another patient was prescribed MMF twice daily. The remaining nonresponder had already been treated with MMF prior to the detection of AAAs. We reduced his dosing interval and replaced the previously employed MMF with cyclosporine. The trough concentrations of these three nonresponders subsequently increased to varying degrees. The serum levels of ADA and AAAs are shown in Table 2. One nonresponder exhibited a significant decrease in the AAA concentration, from 1844.3 ng/mL to 1197.8 ng/mL, after the medication interval was shortened.

**Table 2:** The use of MMF among the 3 nonresponders with detectable serum AAA levels

NR	Before TDM Guidance		Intervention	After TDM Guidance	
	ADA (µg/mL)	AAA(ng/mL)		ADA (µg/mL)	AAA(ng/mL)
1	0.0312	1844.3	Shorten the dosing interval	0.682	1197.8
2	-	701.5	Add MMF	1.238	589.5
3	1.24	578.1	Shorten the dosing interval and change MMF to cyclosporine	1.632	-

NR: nonresponders

MMF: mycophenolate mofetil

Among the 20 patients in the TDM group, three were paediatric patients (15%), aged 13–15 years. Our results showed that 2 of these 3 paediatric patients (66.7% of the paediatric subgroup, 10% of the overall cohort) developed AAAs.

### Establishing the therapeutic window for adalimumab trough concentrations

**Fig. 4:**
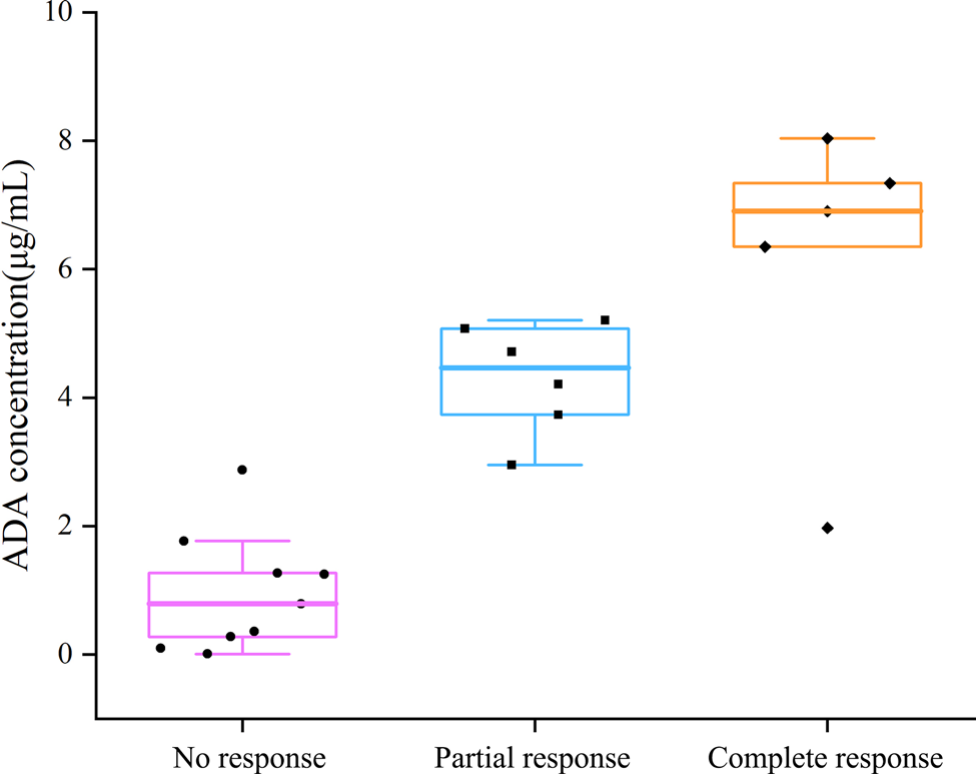
Adalimumab concentration in patients: no, partial, and complete remission

A stratified analysis by clinical response demonstrated significant differences in adalimumab trough concentrations among the CR, PR, and NR groups (Kruskal–Wallis *P* < 0.001; Fig. 4). The median trough concentration was 6.904 (range, 1.968–8.035) µg/mL in the CR group, 4.462 (range, 2.952–5.206) µg/mL in the PR group, and 0.791 (range, 0.010–2.875) µg/mL in the NR group (Table 4). Post hoc pairwise comparisons using the Mann–Whitney U test with Bonferroni correction indicated that the difference between the CR and PR groups did not reach statistical significance. Although the difference between PR and CR did not reach statistical significance, a numerical trend was observed. Therefore, an exploratory analysis was performed by combining PR with NR for comparison against CR, acknowledging the limitation of the small sample size.

**Table 4:** Adalimumab drug levels stratified by clinical response in therapeutic drug monitoring cohort

Clinical response	Adalimumab level, median (min-max), µg/mL	vs. NR	vs. PR	vs. CR
Nonresponse	0.791(0.01–2.875)	—	*P* value = 0.001**	*P* value = 0.006**
Partial response	4.462(2.952–5.206)	*P* value = 0.001**	—	*P* value = 0.378
Complete response	6.904(1.968–8.035)	*P* value = 0.006**	*P* value = 0.378	—

**Fig. 5:**
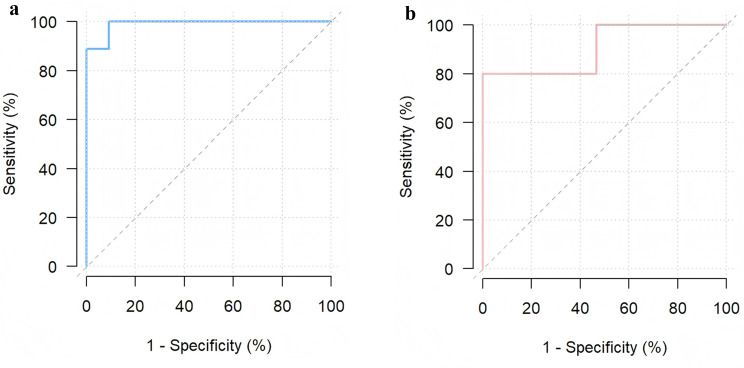
**a** Receiver operating characteristic curves revealing the ability of adalimumab levels to differentiate nonresponse from clinical response. **b** Receiver operating characteristic curves revealing the ability of adalimumab levels to differentiate complete response from partial or nonresponse

ROC curve analysis revealed that a trough concentration > 2.9 µg/mL was associated with any therapeutic response (partial or complete) with 91% sensitivity and 100% specificity (AUC 0.99), whereas a trough concentration > 5.8 µg/mL was associated with a complete response, with 80% sensitivity and 100% specificity (AUC 0.91) (Fig. 5a and b).

### Clinical outcomes of TDM-guided and non-TDM-guided treatment changes

The clinical indicators of patients in both groups improved to varying degrees (Table 3). The BCVA in the TDM group improved from a baseline of (0.34 ± 0.18) LogMAR to (0.11 ± 0.14) LogMAR (*P* < 0.05). The BCVA in the control group without TDM improved from a baseline of (0.35 ± 0.15) LogMAR to (0.25 ± 0.19) LogMAR (*P* < 0.05). The CMT of the TDM group decreased from 337 ± 53.48 μm before treatment to 273.1 ± 19.67 μm after treatment (*P* < 0.05). The CMT in the non-TDM group decreased from 331 ± 33.56 μm before treatment to 295.2 ± 16.45 μm after treatment (*P* < 0.05). In the TDM group, the baseline degree of vitreous inflammation was 3.27 ± 0.70, and after treatment, it decreased to 2.33 ± 0.25 (*P* < 0.05). In the non-TDM group, the baseline degree of vitreous inflammation was 3.43 ± 0.86, and after treatment, it decreased to 2.92 ± 0.28 (*P* < 0.05). At baseline, there was no statistically significant difference in the BCVA, CMT, or degree of vitreous inflammation haze between the two groups (*P* > 0.05). After treatment, there were statistically significant differences in the BCVA, CMT, and degree of vitreous inflammation haze between the two groups (*P* < 0.05).

**Table 3:** Changes in ophthalmic indicators before and after TDM guidance

Group	Number of Eyes	BCVA (${\rm{\bar X \pm S}}$ , logMAR)			CMT (${\rm{\bar X \pm S}}$, µm)			Degree of Vitreous Inflammatory Haze (${\rm{\bar X \pm S}}$)	
		Before Adjustment	After Adjustment		Before Adjustment	After Adjustment		Before Adjustment	After Adjustment
TDM-Guided	40	0.34 ± 0.18	0.11 ± 0.14		337 ± 53.48	273.1 ± 19.67		3.27 ± 0.70	2.33 ± 0.25
*P1*		0.005			0.015			0.003	
Non-TDM-Guided	38	0.35 ± 0.15	0.25 ± 0.19		331 ± 33.56	295.2 ± 16.45		3.43 ± 0.86	2.92 ± 0.28
*P2*		0.002			0.008			0.016	
*P3*		1.000		0.011	0.981		0.029	0.873	0.016

P1 and P2 represent the P-values of the same group after treatment compared to baseline

P3 represents the P-value of two groups at the same time point

*P* < 0.05: There was a statistically significant difference

Moreover, as shown in Table 1, during ADA therapy, the number of inflammation relief days in the TDM group was 398.15 (range 92–801) days, while that in the non-TDM group was 567.1 (range 165–980) days, and the difference was statistically significant (*P* < 0.05). The average recurrence frequency in the TDM group was 1.25 times, while that in the non-TDM group was 2.37 times, and the difference was statistically significant (*P* < 0.05). The average frequency of adverse reactions in the TDM group was 1 occurrence, with a total of 20 occurrences, while the average frequency of adverse reactions in the non-TDM group was 2 occurrences, with a total of 38 occurrences, and the difference was statistically significant (*P* < 0.05).

## Discussion

By integrating serum adalimumab concentrations with clinical responses, we identified a therapeutic trough range of 2.9–5.8 µg/mL in NIU patients, with concentrations above 8 µg/mL suggesting a potential “ceiling effect,” which is consistent with prior evidence in rheumatoid arthritis and psoriasis [33, 34]. On the basis of these thresholds, TDM-guided management achieved superior outcomes compared with empirical adjustment, including faster inflammation control, lower relapse rates, and fewer adverse events in our study. Importantly, TDM led to increased trough concentrations in nonresponders, reduced AAA levels, and increased inflammatory marker levels. Moreover, we innovatively characterized the pharmacokinetic profiles of secondary nonresponders, providing novel insights into adalimumab kinetics in NIU. Collectively, our findings move beyond epidemiological observations of AAAs to establish evidence-based, actionable strategies for TDM in uveitis, bridging the gap between pharmacological monitoring and clinical practice.

The production of AAAs is associated with a lack of response to treatment and reduced ADA persistence. Immunogenicity is one of the main reasons for the loss of response in uveitis patients receiving biologic therapy. The pharmacological effect of ADA involves targeting and blocking the interaction between TNF-α and the cell membrane surface TNF receptors p55 and p75, thereby neutralizing its biological function. AAAs affect the clinical efficacy of ADA by inhibiting its unique subtype (neutralizing antibody) and forming immune complexes (neutralizing and nonneutralizing antibodies) [35]. Current data on AAAs predominantly originate from research in the field of rheumatology [36]. In a three-year prospective study of 272 rheumatoid arthritis patients treated with adalimumab, 28% developed anti-drug antibodies, with most cases emerging within the first 28 weeks of treatment. The presence of these antibodies was strongly associated with substantially reduced serum drug concentrations and poor clinical outcomes [37]. A large prospective cohort study of luminal Crohn’s disease demonstrated that only approximately one-third of patients maintained clinical remission after three years of anti-TNF therapy. Low drug concentrations at week 14 strongly predicted subsequent loss of treatment response, while concomitant immunomodulator use significantly reduced the formation of anti-drug antibodies. AAAs develop in approximately 13% of patients with rheumatoid arthritis, spondyloarthritis, or inflammatory bowel disease who are treated with TNF inhibitors, substantially reducing the clinical response, while concomitant immunosuppressive therapy markedly lowers AAA formation [36]. In summary, the immunogenicity of adalimumab substantially compromises long-term treatment outcomes in patients with autoimmune diseases. In studies of NIU, the reported incidence of AAAs ranges from 7.1% to 52.4% [16, 26, 38], with increasing rates observed over longer treatment durations [16]. AAA-positive patients consistently exhibit significantly lower mean drug concentrations than AAA-negative patients do [18, 26]. Overall, the incidence of AAAs in NIU patients varies widely across studies, and to date, few investigations have provided clinically validated adalimumab concentration thresholds or assessed the real-world effectiveness of TDM in managing NIU.

The development of AAAs is influenced by drug-related, patient-related, and treatment-related factors. Drug-related factors include the degree of antibody humanization [39], molecular structure [40], and drug aggregation [41]. Patient-related factors that may increase the risk of AAA formation include younger age [42], female sex [43], high body mass index (BMI) [26], concomitant systemic immune disorders [21], severe baseline disease activity, long disease duration [44], and specific genetic backgrounds, such as certain HLA alleles [14]. Treatment-related factors, including dosing interval, dose, treatment continuity, and concomitant immunosuppressive therapy, also significantly affect AAA development.

Previous studies have confirmed that age is an important determinant of immunogenicity. In general, younger children are at greater risk of developing immune responses [42], which is consistent with our findings. Multiple factors may contribute to the immunological differences observed between paediatric and adult patients. Children exhibit a higher drug clearance rate [45]. Moreover, because the body surface area-to-weight ratio is greater in children than in adults, linear weight-based dosing (mg/kg) may underestimate the actual drug requirement [46]. In addition, paediatric patients with active IBD or active JIA are often characterized by malnutrition, high inflammatory burden, and hypoalbuminemia, which predispose them to low drug exposure and a vicious cycle of exacerbated immunogenicity [47]. In paediatric patients, younger age and lower body weight (< 10 years or < 30 kg) are associated with an increased risk of immunogenicity, with 10 years of age emerging as a critical threshold [48–51]. Among our 20 patients in the TDM group, three were paediatric patients and 17 were adults. Overall, three patients developed AAAs, including two paediatric patients and one adult patient. The prevalence of AAAs was markedly higher in the paediatric subgroup (66.7%) than in adults. This finding is consistent with previous studies reporting that paediatric patients are more prone to developing AAAs during treatment with TNF-α antagonists than are other age groups [48–51]. In our study, however, the paediatric subgroup consisted of adolescents older than 10 years and weighing more than 30 kg, whose pharmacokinetic profiles are more comparable to those of adults. Therefore, we combined the paediatric and adult patients in the current analysis. We acknowledge that combining paediatric and adult patients may have partly obscured age-related immunological differences. Given the limited sample size of the present study, expanding the cohort is essential to better characterize data patterns and strengthen the robustness of our findings. Future studies with larger paediatric cohorts are particularly warranted to enable age-stratified analyses and to further clarify the impact of age on immunogenicity and treatment outcomes.

Current evidence indicates a substantially greater risk of AAA development in patients with uveitis associated with systemic diseases than in those with idiopathic cases, as supported by numerous clinical studies. Cordero-Coma et al. identified systemic comorbidities—including spondyloarthritis, juvenile idiopathic arthritis (JIA), and sarcoidosis—in 62.5% (5/8) of AAA-positive patients [24]. In contrast, Eurelings et al. reported AAA incidence rates of 67% (4/6) in JIA-associated uveitis patients compared with 48% (11/23) in idiopathic cases [52]. This association was further strengthened by the findings of Albert et al., who reported that 91.7% of AAA-positive patients had underlying systemic diseases, whereas only 42.1% of AAA-negative patients did (*P* < 0.01) [53]. During this study, of the three AAA-positive patients, two had underlying systemic diseases: one had JIA and the other had Behçet’s disease. The underlying mechanism may be attributed to the heightened immune activation state and more profound immune dysregulation characteristic of systemic diseases, which predisposes these patients to stronger immune responses against foreign protein therapeutics, consequently increasing the risk of anti-drug antibody formation. Additionally, the presence of autoantibodies such as antinuclear antibodies has been clinically correlated with both increased AAA formation and reduced treatment efficacy, suggesting a potential synergistic mechanism between preexisting autoimmune profiles and antidrug antibody development [43].

The weekly maintenance dose of ADA is associated with a reduced risk of AAA development. For patients receiving standard weekly dosing, increasing the frequency to weekly has been demonstrated to be an effective strategy for managing refractory ocular inflammation; however, its correlation with serum AAA levels has not been investigated [54]. Increasing the weekly dosage is also an effective strategy for patients with rheumatoid arthritis and ulcerative colitis who do not respond to ADA, as evidenced by studies by Bartelds et al. [55], Wolf et al. [56], and Bartelds et al. [57], and similar conclusions have been reached in studies focusing on NIU [26]. In our study, after a 6-week period of shortened dosing intervals, the measured concentration of AAAs in one patient decreased from 1.8443 µg/mL to 1.1978 µg/mL, while the ADA trough level increased from 0.0312 µg/mL to 0.682 µg/mL.

In NIU, the concomitant use of conventional immunosuppressive agents, such as MTX or MMF, has been shown to significantly reduce the development of AAAs, thereby improving drug retention and the clinical response. Francesco Pichi et al. confirmed that for NIU patients with low adalimumab drug concentrations and inadequate immune responses, increasing the adalimumab injection dose combined with adding low-dose methotrexate represents the most effective adjustment strategy to reduce the occurrence of anti-drug antibodies [26]. Leinonen et al. discovered in their study of 31 patients with JIA-related uveitis that AAAs occurred more frequently in patients not receiving methotrexate concurrently [58]. Previous studies on rheumatoid arthritis, spondyloarthritis, psoriasis, inflammatory bowel disease, and Crohn’s disease have consistently demonstrated that concomitant immunosuppressant use reduces anti-drug antibody formation and improves clinical outcomes [30, 59]. Mechanistically, immunosuppressants suppress autoreactive B cell activation and differentiation, limiting antibody generation against adalimumab [60]. They also attenuate the activity of T helper cells, particularly T follicular helper (Tfh) cells, thereby disrupting germinal centre responses required for high-affinity anti-drug antibody formation [61]. Collectively, these mechanisms provide a robust biological basis for combining adalimumab with conventional immunosuppressants in NIU management to mitigate immunogenicity and optimize long-term therapeutic outcomes. Our findings also confirm this point; after a 6-week course of combined treatment with mycophenolate mofetil, the measured concentration of AAAs in one patient decreased from 0.7015 µg/mL to 0.5895 µg /mL.

Multiple studies have confirmed that patients carrying the HLA-DQA105 allele exhibit a higher risk of immunogenicity during treatment with tumour necrosis factor-alpha (TNF-α) inhibitors, such as adalimumab and infliximab [62, 63]. However, current evidence has not yet reached a consensus on the association between this gene and a diminished treatment response as well as the incidence of adverse events [64]. The potential mechanistic role of the HLA-DQA105 allele in AAA formation was not systematically investigated in this study. Subsequent research will focus on in-depth exploration of the molecular biological associations between this genotype and the development of immunogenicity.

TDM of TNF-α inhibitors is well established in immune-mediated diseases, with guidelines such as the 2017 AGA recommendations for IBD [65] and accumulating evidence confirming its benefit in cases of nonresponse or loss of response [66–70]. More recently, the 2022 EULAR task force outlined key considerations for TDM in rheumatoid arthritis and recommended passive TDM to optimize treatment strategies [33]. However, in patients with NIU receiving TNF-α inhibitors, there are no established guidelines and there is no expert consensus on the role of TDM or on the optimal timing for measuring serum levels of ADA and AAAs, although some studies suggest that it may be beneficial. Sejournet et al. reported that in patients with NIU, adalimumab trough levels were significantly higher in responders than in nonresponders. TDM allowed clinical improvement in 87% of nonresponders, and 48.4% of responders were able to reduce their adalimumab dose without disease relapse, with 80% of patients maintaining remission [18]. Bellur et al. found that 35.7% of NIU patients who received adalimumab tested positive for AAAs, which was associated with significantly lower drug concentrations. They also proposed a clinical response threshold of 2.7 µg/mL for adalimumab or an AAA level below 15.2 µg/mL [27]. In summary, studies of NIU have consistently shown that the presence of AAAs is associated with substantially reduced adalimumab levels and poorer clinical outcomes, providing informative reference ranges for adalimumab trough concentrations, with suggested lower efficacy thresholds ranging from approximately 2.70 to 7.95 µg/mL [26–28]. However, due to differences in assay methodologies, patient populations, and other confounding factors, the proposed optimal thresholds have been inconsistent across studies. Therefore, we aimed to define an optimal therapeutic threshold range for our study cohort by integrating clinical responses with the adalimumab serum concentration, which was determined to be 2.9–5.8 µg/mL, and to guide TDM based on this range. Admittedly, given the limited sample size, our investigation of threshold determination remains exploratory, and its accuracy and robustness require confirmation in larger, prospective studies.

Among 20 NIU patients managed with TDM guidance, the mean serum adalimumab (ADA) concentration increased from 2.88 ± 1.95 µg/mL (range: 0–6.94 µg/mL) to 4.5 ± 3.07 µg/mL (range: 0.068–11.036 µg/mL) (Fig. 2). Furthermore, we innovatively characterized the pharmacokinetic profiles of six secondary nonresponders over 14 days following a single ADA dose. Most patients reached the peak ADA concentration between days 3 and 10, with a mean peak at approximately days 5–8. Considerable inter-patient variability in concentration-time profiles was observed, and the peak levels were generally low; 66.7% (4/6) of patients had peaks below the identified therapeutic threshold of 2.9–5.8 µg/mL. Among these six secondary nonresponders, three patients (50%) were positive for AAAs (1041 ± 570 ng/mL), and they exhibited overall lower ADA concentrations, consistently below 2.9 µg/mL. The trough ADA level in secondary nonresponders was significantly lower than that in responders (0.55 µg/mL vs. 3.43 µg/mL, *p* < 0.001), and it increased to 1.22 µg/mL following TDM-guided adjustments. In the three AAA-positive nonresponders, TDM interventions—including shortened dosing intervals and modification of concomitant immunosuppressive therapy—reduced the AAA titre ADA trough level. Collectively, these findings indicate that the presence of AAAs is associated with lower mean ADA concentrations and that TDM-guided dose optimization can reduce AAA levels, restore effective ADA exposure, and improve clinical outcomes. However, inter-individual metabolic variations among patients may lead to fluctuations in serum drug concentrations. Moreover, the appropriateness of this specific time point as a reliable indicator for the minimum trough concentration reflecting steady-state drug exposure remains a subject of debate. Furthermore, there are limitations to short-term pharmacokinetics analysis. Given the long half-life of ADA, which is approximately 10–20 days, its clinical efficacy is typically demonstrated during the long-term maintenance phase, such as every 2 weeks over a period of at least 12 weeks. Thus, fluctuations in concentration 14 days following a single administration may not be directly indicative of short-term drug reactions, but they could offer indirect insights into long-term efficacy.

As depicted in Fig. 2, three nonresponders had adalimumab trough concentrations exceeding 8 µg/mL; however, their clinical symptoms did not improve further, consistent with prior observations in rheumatoid arthritis and psoriasis patients, where higher trough levels beyond a certain threshold confer no additional therapeutic benefit, indicating the presence of a treatment plateau [33]. This may directly lead to two drawbacks: (1) Increased infection risk: the incidence of severe infections increases when the ADA concentration exceeds 8 µg/mL [71, 72]. (2) Increased medical expenses: Maintaining ADA concentrations beyond the treatment window increases annual treatment costs without yielding additional benefits. Therefore, for patients who have achieved disease stability and exhibit adalimumab trough concentrations exceeding 8 µg/mL, dose reduction may be considered, with careful monitoring for potential adverse effects.

## Conclusion

In our study, a therapeutic trough concentration range of 2.9–5.8 µg/mL was preliminarily defined for adalimumab in NIU patients, and the results highlighted that levels above 8 µg/mL may result in a potential “ceiling effect” that may result in adverse events, which should be avoided. The development of anti-adalimumab antibodies was closely associated with poorer clinical outcomes. Moreover, optimizing adalimumab therapy based on TDM resulted in better clinical outcomes, reduced antibody levels, and increased drug exposure compared with those of empirical management, consistent with findings in other immune-mediated inflammatory diseases [73, 74]. By measuring serum trough levels and antibody status, individualized treatment can be implemented, preventing overtreatment and substantially reducing healthcare costs, thereby underscoring the clinical and economic value of TDM in NIU management. However, this study has several obvious limitations. First, the single-centre, retrospective design and relatively small sample size may limit the generalizability of our findings. Second, the inclusion of both paediatric and adult patients in a single analysis may have masked age-related immunologic differences; future studies with sufficient paediatric representation are warranted to enable age-stratified analyses and clarify the impact of age on immunogenicity and treatment outcomes. Third, there is a lack of standardized consensus on the optimal blood sampling time for TDM of ADA. In this study, blood samples were collected within 4 h prior to the subsequent ADA administration, but the appropriateness of this specific time point as a reliable indicator for the minimum trough concentration reflecting steady-state drug exposure remains a subject of debate. Finally, although our dual-threshold model appears promising, the findings should be regarded as exploratory given the limitation of the sample size. Moreover, its clinical applicability remains to be established, as prospective, multicenter studies are still needed to validate its effectiveness across diverse patient populations. Nevertheless, despite these limitations, our findings provide valuable insights into the complex management of refractory uveitis. The quantification of drug and antibody levels may offer clinically actionable data to guide personalized ADA therapy, enabling more rational and sustainable treatment strategies.

## Data Availability

The raw data supporting the conclusions and the materials used in this study, including reagents and protocols, are available from the corresponding author upon request.

## Abbreviations

TDM: Therapeutic drug monitoring
NIU: Non-infectious uveitis
ADA: Adalimumab
AAA: Anti-adalimumab antibody
TNF-α: Tumor necrosis factor alpha
IBD: Inflammatory bowel disease
BCVA: Best-corrected visual acuity
SUN: Standardization of Uveitis Nomenclature
AC: Anterior chamber
SS-OCTA: Swept-source optical coherence tomography angiography
FFA: Fluorescein angiography
ICGA: Indocyanine green angiography
cDMARDs: Conventional disease-modifying antirheumatic drugs
MTX: Methotrexate
AZA: Azathioprine
MMF: Mycophenolate mofetil
VKH: Vogt-Koyanagi-Harada disease
JIA: Juvenile idiopathic arthritis
CMT: Central macular thickness
EULAR: European League Against Rheumatism
